# État des lieux des échographies obstétricales au service d’imagerie du Centre Hospitalier Universitaire Bogodogo du 1^er^ janvier 2016 au 31 décembre 2018

**DOI:** 10.11604/pamj.2021.38.286.21353

**Published:** 2021-03-18

**Authors:** Bénilde Marie Ange Tiemtoré-Kambou, Aischa Madina Napon, Tounougma Kaboré, Nina Astrid Ndé Ouédraogo, Lisa Kéré Nidjergou, Mohammed Tall, Issouf Franck N’dama Sieba, Abel Bamouni, Ousséini Diallo, Rabiou Cissé

**Affiliations:** 1 Service d’Imagerie Médicale et de Radiologie Interventionnelle, Centre Hospitalier Universitaire Bogodogo, Ouagadougou, Burkina Faso,; 2 Service de Radiologie, Centre Hospitalier Universitaire Pédiatrique Charles de Gaulle, Ouagadougou, Burkina Faso,; 3 Service de Radiologie, Centre Hospitalier Universitaire Yalgado Ouédraogo, Ouagadougou, Burkina Faso

**Keywords:** Bilan, échographie obstétricale, qualité, Status, obstetric ultrasound, quality

## Abstract

L´échographie obstétricale réalisée selon les normes depuis sa prescription jusqu´au compte rendu est un gage de qualité. Le but de notre étude était de faire le point des échographies obstétricales en vue de rendre rationnelle les échographies de suivi de la grossesse. Il s´est agi d´une étude transversale descriptive avec collecte rétrospective des données à travers les comptes rendus d´échographies obstétricales réalisées du 1^er^ janvier 2016 au 31 décembre 2018. Sur les 13487 échographies de la période d´étude, 2355 étaient des échographies obstétricales, constituant 17,46% de l´activité échographique. L´âge moyen des gestantes était de 27,54 ans (±6,19). Le CHU de Bogodogo était la structure demandeuse pour 86,88%. Les paramédicaux étaient prescripteurs dans 66,47%. Les échographies du troisième trimestre constituaient 57,06%. L´âge gestationnel dans 12,99% était supérieur à 37 semaines tandis que dans 66,37% l´âge gestationnel était supérieur à 24 semaines d’aménorrhées (SA). Le suivi prénatal était indiqué dans 54,48%. Les grossesses étaient évolutives dans 97,49%. Les grossesses gémellaires représentent 2,72% et les grossesses pathologiques échographiques 11,80%. Le Doppler obstétrical a été réalisé dans 2,12% avec la pré-éclampsie comme indication principale (52%). Il y avait 1,18% de malformation avec atteinte du système nerveux dans 60,71%. A travers cet état des lieux, la formation des prescripteurs de l´échographie obstétricale apparait primordiale. La mise en place d´un observatoire des pratiques en échographie obstétricale serait d´un atout pour la qualité de ces examens médicaux.

## Abstract (en)

Obstetric ultrasound performed according to standards from its prescription to the report is a guarantee of quality. The aim of our study was to take stock of obstetrical ultrasounds in order to make rational the ultrasound follow-ups of pregnancy. This was a descriptive cross-sectional study with retrospective data collection through the reports of obstetrical ultrasounds performed from the 1^st^ January 2016 to 31^st^ December 2018. Of the 13,487 ultrasounds in the study period, 2,355 were obstetric ultrasounds, constituting 1746% of the ultrasound activity. The mean gestational age was 27.54 years (± 6.19). The CHU Bogodogo was the requesting structure for 86.88%. Paramedics were prescribers in 66.47%. Third trimester ultrasounds made up 57.06%. Gestational age in 12.99% was greater than 37 weeks while in 66.37% gestational age was greater than 24 weeks. Prenatal follow-up was indicated in 54.48%. Pregnancies were progressive in 97.49%. Twin pregnancies represent 2.72% and ultrasound pathological pregnancies 11.80%. Obstetric Doppler was performed in 2.12% with pre-eclampsia as the main indication (52%). There was 1.18% malformation with nervous system involvement in 60.71%. Through this inventory, the training of prescribers of obstetric ultrasound appears essential. The establishment of an observatory of obstetric ultrasound practices would be an asset for the quality of these medical examinations.

## Introduction

L´échographie obstétricale est un examen médical permettant d´évaluer le bien-être du fœtus et de contribuer à une bonne prise en charge de la grossesse [1]. Elle obéit à des règles depuis sa prescription et ses résultats pour être d´un apport certain dans la prise en charge de la grossesse [2]. Devant l´extension de l´hôpital passant d´un hôpital de district à un Centre Hospitalier Universitaire, il nous est paru important de marquer un arrêt sur cet examen et d´en dégager les écueils pour pouvoir l´améliorer tant au niveau des prescripteurs que des réalisateurs. Le but de notre étude était de réaliser un bilan des échographies obstétricales dans le service d´imagerie médicale et radiologie interventionnelle du CHU Bogodogo de 2016 à 2018. Cela à travers la détermination de la fréquence des échographies obstétricales et les caractéristiques sociodémographiques des patientes. Puis nous avons déterminé les indications des prescriptions d´échographies obstétricales, les qualifications du personnel et décrit les résultats des échographies obstétricales.

## Méthodes

Il s´est agi d´une étude transversale descriptive avec collecte rétrospective des données du 1^er^ janvier 2016 au 31 décembre 2018. La population d´étude était composée de toutes les femmes enceintes ayant réalisé une échographie obstétricale dans le service d´imagerie médicale et de radiologie interventionnelle durant la période d´étude. Les femmes dont les comptes rendus d´échographies étaient incomplets ont été exclues. La collecte des données a été réalisée à l´aide d´une fiche de collecte individuelle remplie à partir des comptes rendus d´échographie obstétricales. Ces échographies ont été réalisées au cours de la première année avec un appareil d´échographie Mindray DC 8 Expert mis en service en mars 2012 et les deux autres années avec deux appareils, un Siemens NX 300 mis en service en mars 2017 s´étant ajouté au parc échographique du service.

Les variables étudiées étaient: les caractéristiques sociodémographiques comprenant l´âge des gestantes; la structure demandeuse de l´examen; les qualifications du personnel médical impliqué à savoir les prescripteurs et les échographistes; les indications des échographies obstétricales en fonction des termes; les résultats des échographies obstétricales réalisées. Les données ont été saisies et analysées grâce au logiciel Epi Info dans sa version 7.2 et au logiciel Excel 2016. Avec ces données secondaires puisque utilisant des comptes rendus, nous avons obtenu une autorisation de collecte auprès des responsables du CHU de Bogodogo. Les résultats étaient anonymes.

## Résultats

Notre étude a porté sur 2355 comptes rendus d´échographies dont 374 pour l´année 2016, 1035 pour l´année 2017 et 955 pour l´année 2018. Au cours de l´année 2016 il y avait un total de 1537 échographies dont 24,33% d´échographies obstétricales. En 2017 il y avait un total de 7213 échographies soit 14,34% échographies obstétricale et en 2018 il y avait 4737 échographies soit 20,16% échographies obstétricales. Au cours de la période d´étude les échographies obstétricales constituaient 17,46% de l´activité échographique du service. L´âge moyen des patientes était de 27,54 ans (± 6,19) avec des extrêmes de 12 ans et 51 ans. Les tranches d´âge les plus représentées étaient par ordre croissant celle de [30-34] avec 21,31% (502), suivie de la tranche de [20-24] avec 25,64% (604) et celle de [25-29] avec 28,42% (669) des femmes. Le Centre Hospitalier Universitaire de Bogodogo était la structure demandeuse dans 86,88% (2046) des cas. Les Centres de Santé et de Promotion Sociale (CSPS) et les Centres Médicaux avec Antenne chirurgicale (CMA) étaient représentés à 7,56% (178). Les structures de santé privée constituaient 2,17% (51) des centres d´où provenaient la demande. Dans 80 cas, la structure demandeuse n´était pas précisée. La qualification des prescripteurs était précisée dans 14,05% (331) des comptes rendus. L´échographie était prescrite dans 66,47% (220) par des paramédicaux (SFE/infirmier) contre 33,53% (111) prescrites par des médecins. Les échographistes dans notre étude étaient uniquement des médecins spécialistes en imagerie au nombre de quatre. Le nombre d´année d´expérience en imagerie médicale variait de 2 à 15 ans. Un de 15 ans, 1 de 10 ans et les deux autres de 2 ans.

**Figure 1:**
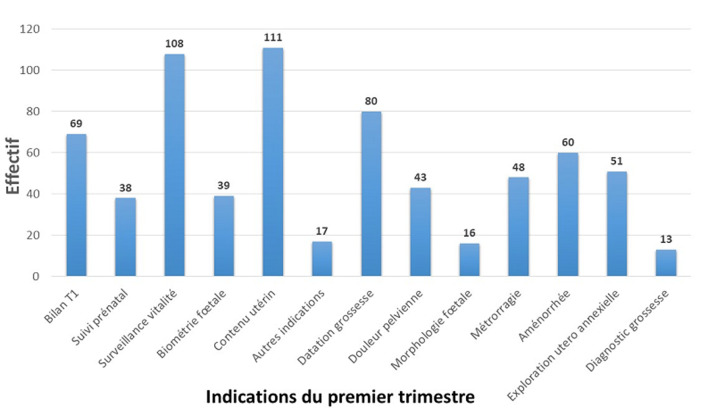
répartition des indications de l´échographie obstétricale au premier trimestre

**Figure 2:**
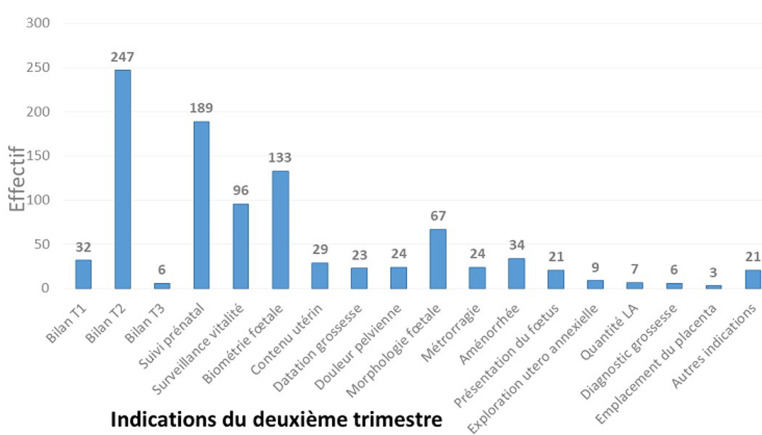
répartition des indications de l´échographie obstétricale au deuxième trimestre

**Figure 3:**
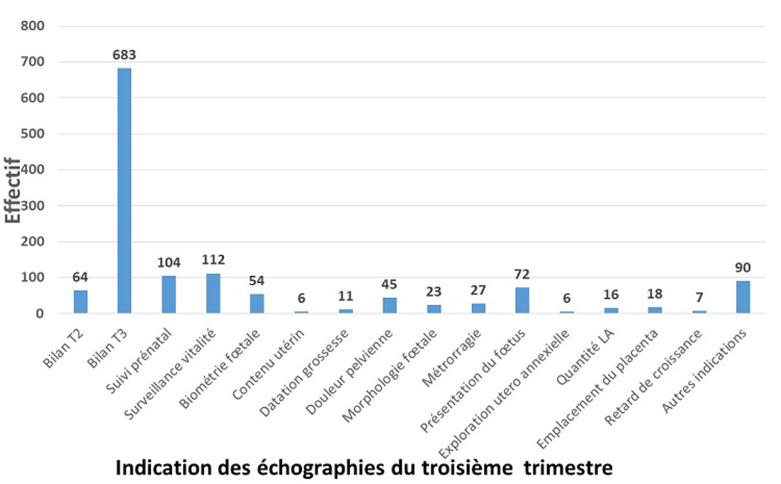
répartition des indications de l´échographie obstétricale au troisième trimestre

**Figure 4:**
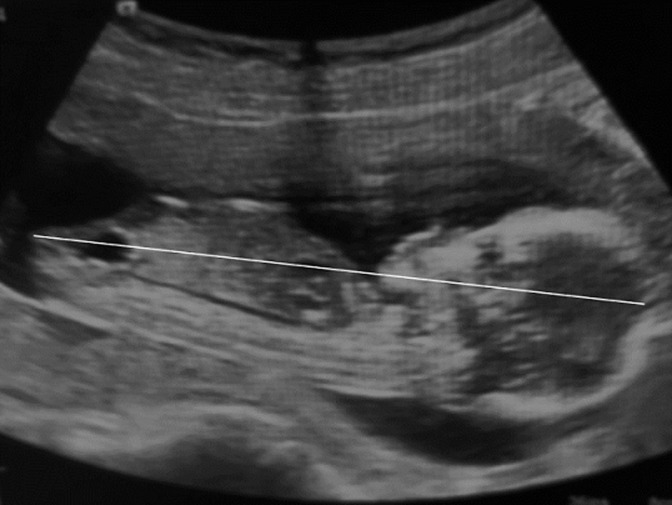
mesure de la longueur cranio caudale: embryon de 13 semaines 3 jours à l´échographie 2D

**Figure 5:**
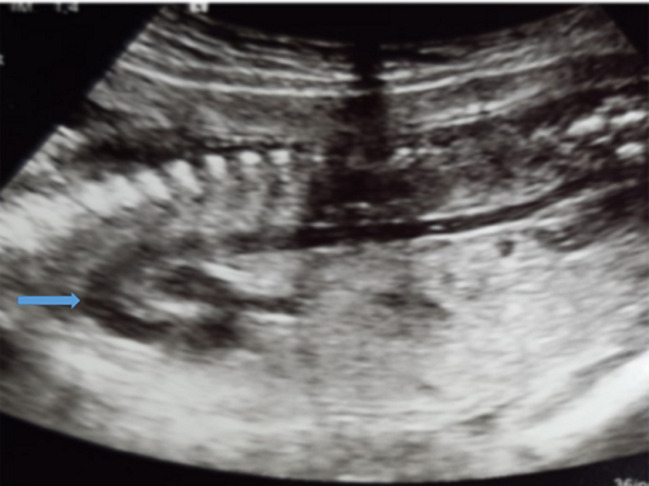
échographie 2D montrant une crosse aortique (flèche) chez un fœtus de 22 semaines

Les indications des échographies sont représentées par les indications des échographies du premier trimestre (Figure 1), les indications des échographies du deuxième trimestre (Figure 2) et celles des échographies du troisième trimestre (Figure 3). Au premier trimestre de grossesse 334 échographies étaient réalisées soit 14,18%. La Figure 4 montre une coupe d´un embryon de 13 semaines 3 jours pour la mesure de la longueur cranio-caudale. Au deuxième trimestre 683 échographies étaient réalisées soit 29%. La Figure 5 montre une coupe de la crosse aortique chez un fœtus de 22 semaines et les échographies du troisième trimestre étaient de 1338 soit 56,81%. Dans 66,36% (1563) des échographies l´âge gestationnel était supérieur à 24 semaines et dans 12,99% (306) supérieur à 37 semaines. Les grossesses arrêtées représentaient 2.29% (54) des grossesses pour 97,71% (2301) de grossesses évolutives. La tranche de 7-12 SA était la plus fréquente avec 22 cas soit 40,74%. La grossesse était monofœtale dans 97,28% (2291) contre 2.72% (64) de grossesse gémellaire; elles étaient bichoriales biamniotiques et monochoriales biamniotiques dans chacune 48,44% des cas.

**Figure 6:**
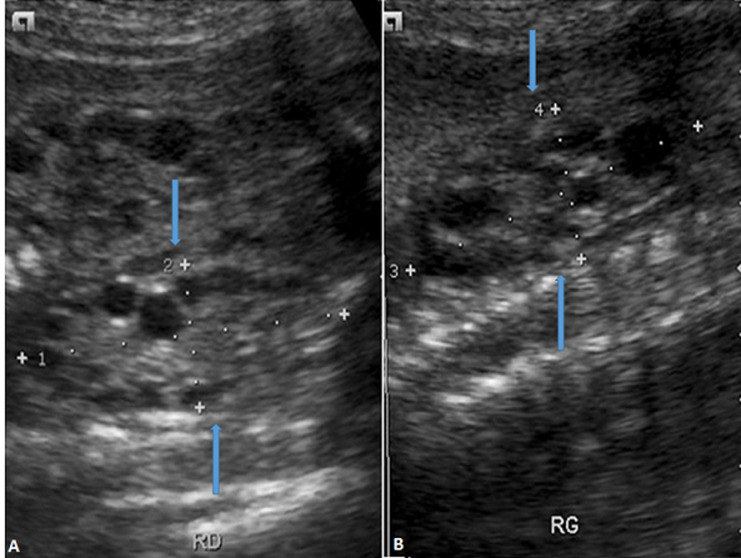
A) polykystose rénale bilatérale (droite flèche); B) (gauche flèche) chez un fœtus de 30 semaines; les reins sont vus en coupe longitudinale à l'échographie 2D

Le Doppler échographique représentait 2,12% (50) des échographies obstétricales. Parmi ces échographies Doppler, 10% (5) étaient réalisées à la demande du prescripteur contre 90% (45) préconisées par le radiologue. L´échographie Doppler était fréquemment réalisée à partir de 30 SA soit 78% (39). Les indications pour raison de pré-éclampsie étaient les plus fréquentes soit 52% (26). Le Doppler était normal dans 64 % (32) des cas puis venaient les souffrances fœtales à 16% et le retard de croissance intra-utérin (RCIU) à 8%. Nous avons retrouvé 1,18% (28) de malformations. Les malformations congénitales étaient diagnostiquées au troisième trimestre soit 85,70%. Les indications étaient dominées par le bilan du troisième trimestre à 32,14%, suivies des indications pour confirmation de malformations avec 17,86%. Les malformations cranio-encéphaliques étaient les plus fréquentes avec 60,71% (17) suivies de l´appareil uro-génital avec 35,71% (10). La Figure 6 montre deux reins en coupe longitudinale avec une polykystose chez un fœtus de 30 semaines. Les polymalformations dans notre série représentaient 14,29% (4) pour 85,71% (24) de mono malformations. Dans 4,04% (54) d´échographie, la présentation était vicieuse au troisième trimestre de grossesse pour 95,96% (1284) de présentation céphalique.

## Discussion

Notre étude étant une étude rétrospective et dans le seul centre d´imagerie du CHU; il existait un biais de sélection. L´archivage des données nous soumettait à un biais d´information du fait de la non complétude des comptes rendus. Le taux d´échographie obstétricale par rapport aux autres types d´échographies correspond en moyenne au 1/5 de l´activité échographique totale. Cela s´expliquerait par le fait qu’avec l´ouverture progressive de certains services spécialisés, les demandes étaient multiformes et ne concernaient pas seulement l´obstétrique comme lors de l´année 2016 où les échographies obstétricales correspondaient au quart des échographies. L´échographie était prescrite dans 66,47% des cas par des paramédicaux contre 33,53% par des médecins. Ces résultats sont proches de ceux trouvés par Dagnan *et al*. [3] qui trouvait que l´examen échographique était demandé en majorité par les sages-femmes 66,3% puis les médecins à 20,68%. La sage-femme se trouve en permanence au cœur des problèmes de maternité [4]. D´où la proportion élevée des sages-femmes/infirmières pour les prescripteurs de l´échographie obstétricale car les premiers agents de santé en contact des gestantes [5]. La fréquence des indications des échographies obstétricales varie en fonction du trimestre de grossesse. Au premier trimestre elle n´est pas souvent prescrite en absence de signe d´appel, tandis qu´elle est prescrite dans 98,5% par les praticiens au troisième trimestre [6]. Les indications les plus fréquentes au premier trimestre de la grossesse étaient dominées par l´étude du contenu utérin qui représentait 16,01%, la vitalité de l´embryon 10,75% et la datation de la grossesse 7,96%.

Nos résultats diffèrent de ceux trouvés par Utoo *et al*. [5] avec 21% d´indication de bien-être de l´embryon, 6% de vitalité de l´embryon, 4,8% de diagnostic de grossesse et 3,2% de datation de la grossesse. Certaines indications ne sont pas conformes notamment les indications de l´échographie du premier trimestre telles la « biométrie » et les indications « non argumentées » et/ou incomplètes telles que l' « étude utero-annexielle » et l' « aménorrhée ». Au deuxième trimestre les indications diffèrent de celles trouvées par Essiben *et al*. [6] où 93,4% des prescriptions l´étaient pour l´étude de la morphologie fœtale. Certaines indications témoignent de la méconnaissance par les prescripteurs des indications de l´échographie selon la situation clinique (présentation du fœtus, diagnostic de grossesse, bilan de T3). D´où la recommandation du Collège Français d'Échographie Fœtale (CNEOF) qui passe par une formation des demandeurs des échographies obstétricales et fœtales, avec le double objectif de potentialiser l´intégration des données dans leur pratique clinique et de renforcer leur connaissance des critères de qualité des examens [7]. Pour les échographies du troisième trimestre, 56,02% étaient demandées pour faire le bilan du troisième trimestre de la grossesse, suivie de la vitalité 9,15%, le suivi prénatal à 8,56% et la présentation du fœtus à 5,87%. Ces résultats sont en déphasage avec ceux retrouvés par Essiben *et al*. [6] où 75,4% d´échographies étaient prescrites au troisième trimestre pour apprécier la croissance fœtale. Nos résultats sont différents aussi de ceux de Utoo *et al*. [5] avec 21% d´indications pour le bien-être fœtal, 6% pour la surveillance de la vitalité fœtale. Les échographies du troisième trimestre étaient les plus fréquentes avec un pourcentage de 57,06%. Ces résultats sont proches de ceux trouvés par Swanson *et al*. [8] qui trouvait 59% des échographies obstétricales réalisées au troisième trimestre contre 46,1% par Dagnan *et al*. [3]. Ces résultats s´expliqueraient par un début tardif des consultations prénatales.

En outre 66,37% des échographies étaient réalisées après 24 SA. Les recommandations de l´OMS 2016 préconisent au moins une échographie avant 24 semaines de grossesse [3]. Les malformations fœtales ont été retrouvées chez 1,18% des femmes réalisant une échographie obstétricale. Ce taux est comparable à celui de Guena *et al*. [9] où les malformations fœtales ont été diagnostiquées dans 1,21% des cas. Ce résultat est différent de celui de N´Timon *et al*. [10] chez qui les fœtus malformés représentaient 0,83%. La prévalence des malformations diffère d´une étude à une autre. Elle pourrait s´expliquer par les différences d´échantillonnage, la durée des études et des critères d´inclusion. La prévalence et le type des malformations sont différents d´un pays à un autre, et dans le même pays, ils diffèrent d´une région à l´autre. Cela peut suggérer le rôle de l´environnement, des variations génétiques et ethniques qui pourraient être des facteurs de risque. Ces malformations constituent, en plus de leur répercussion physiologique et psychologique sur l´individu malade, un fardeau social et économique pour les parents et la société entière [9]. Les malformations ont été diagnostiquées dans 85,70% des cas au troisième trimestre. Ces résultats sont différents de ceux trouvés par Kehila *et al*. [11]. En effet dans leur série, la majorité des malformations (72%) ont été détectées au deuxième trimestre. Cette différence s´expliquerait d´une part par la réalisation tardive de la première échographie obstétricale dans notre contexte. L´accent doit être mis ici sur le respect de la check-list de réalisation des échographies morphologiques au deuxième trimestre pour ne pas occulter une anomalie et pour répondre à une des recommandations du CNEOF qui est la mise en place d´un observatoire des pratiques en échographie obstétricale et fœtale [7]. Le taux de monomalformations de 85,71% s´apparente à ceux de N´Timon *et al*. [10]. Les monomalformations prédominaient dans 82,81% cas. Le système nerveux était le plus affecté 60,71% comme chez N´Timon dans 39,74% suivi de l´appareil uro-génital 30,77% et chez Guena *et al*. [9] dans 46,34%. Dans toutes ces différentes études, l´atteinte du système nerveux pourrait être due à une mauvaise supplémentation en acide folique ou aux causes hormonales comme le diabète [12,13].

## Conclusion

L´échographie obstétricale permet une surveillance de la grossesse. Elle est en majorité prescrite par les paramédicaux. Les différentes indications n´étaient pas adaptées au terme. Les grossesses du troisième trimestre étaient les plus nombreuses. Ainsi la mise en place d´un observatoire des pratiques en échographie obstétricale et fœtale et une formation des demandeurs des échographies obstétricales et fœtales, avec le double objectif de potentialiser l´intégration des données dans leur pratique clinique et de renforcer leur connaissance des critères de qualité des examens seraient des gages de qualité de ces échographies. Cette qualité nous permettrait de faire de la grossesse une expérience positive comme le recommande l´OMS.

### Etat des connaissances sur le sujet

- L´état des prescriptions d´échographies obstétricales hors contexte: au Burkina Faso, moins de 25% des femmes parturientes font des échographies durant leur grossesse;
- L´état des comptes rendus d´échographie obstétricales hors contexte. Les recommandations de l´OMS 2016 préconisent au moins une échographie avant 24 semaines de grossesse;
- L´apport de l´échographie obstétricale dans la surveillance de la grossesse et de la réduction de la mortalité maternelle et fœtale. Trois échographies sont conseillées par le Collège français d'échographie fœtale (CFEF): premier examen: 11-13 SA (si possible par voie endovaginale) datation et vitalité fœtale; 2^e^ examen: entre 22 et 24 SA = dépistage des malformations; 3^e^ examen: date = 31-33 SA croissance fœtale.

### Contribution de notre étude à la connaissance

- Les paramédicaux sont les principaux prescripteurs d´échographie obstétricale alors que ceux-ci ne les réalisent pas: l´échographie était prescrite dans 66,47% des cas par des paramédicaux contre 33,53% par des médecins;
- Le respect des règles de prescription de cet examen médical n´est pas atteint: l´échographie obstétricale est prescrite dans 98,5% par les praticiens au troisième trimestre;
- Les échographies sont réalisées tardivement après 24 semaines: le nombre d´échographies obstétricales réalisées après 24 semaines était de 66,37%.
